# Enhanced Tumor Targeting and Radiotherapy by Quercetin Loaded Biomimetic Nanoparticles

**DOI:** 10.3389/fchem.2020.00225

**Published:** 2020-03-31

**Authors:** Chunyu Huang, Tongkai Chen, Daoming Zhu, Qinqin Huang

**Affiliations:** ^1^Department of Molecular Pathology, The Second Affiliated Hospital of Zhengzhou University, Zhengzhou, China; ^2^Key Laboratory of Artificial Micro- and Nano-Structures of Ministry of Education, School of Physics and Technology, Wuhan University, Wuhan, China; ^3^Science and Technology Innovation Center, Guangzhou University of Chinese Medicine, Guangzhou, China

**Keywords:** cancer cell membrane, quercetin, mesoporous silicon nanoparticles, drug delivery, enhanced radiotherapy

## Abstract

In Chinese traditional medicine, quercetin (QT) plays a fundamental role in the treatment of asthma, as an anti-allergen and to lower blood pressure. Recent evidence suggests that QT can improve tumor radiosensitivity through multiple mechanisms. However, poor tumor tissue targeting ability and low water solubility of QT limit its usefulness in the treatment of cancers. Herein, we designed a novel drug delivery system (CQM) consisting of inner QT loaded mesoporous silica nanoparticles (MSNs) and outer cancer cell membranes (CM). The developed nanoplatform had strong anti-cancer effects under X-ray irradiation and good QT loading characteristics. In addition, CQM effectively targeted tumor tissues. Results of *in vitro* and *in vivo* experiments demonstrated that the developed CQM drug delivery system has excellent tumor targeting ability and effectively inhibited tumor growth. Therefore, the CQM platform realized targeted drug delivery and radiotherapy sensitization, which provided a newfangled idea of cancer treatment.

## Introduction

In recent years, a number of novel cancer therapies have been developed such as photo thermal therapy (Lin et al., 2017; Yu et al., 2017; Zhou et al., 2018; Cao et al., 2019; Liu et al., 2019; Yang et al., 2019), photodynamic therapy (Song et al., 2015; Sun et al., 2017; Jiang et al., 2018), and chemo dynamic therapy (Zhang et al., 2016, 2019; Ma et al., 2018; Hu et al., 2019; Lei et al., 2019; Li et al., 2019; Xia et al., 2019; Xie Z. et al., 2019), however their adoption has been limited by unstable clinical effects. A traditional treatment for cancer, radiotherapy, has been extensively applied in clinical settings. Radiotherapy promotes the production of reactive oxygen species in tumor tissues, thus promoting tumor cell apoptosis and inhibition of tumor growth (Liu et al., 2015; Song et al., 2016a,c; Du et al., 2017; Lyu et al., 2019). Radiotherapy however, does not discriminate between normal and tumor cells, furthermore a number of tumor types are intrinsically resistant to radiotherapy which has led to a growing need for improved cancer therapies (Fan et al., 2015; Song et al., 2016b, 2017). Therefore, there is an urgent need to develop allergens in radiotherapy to enhance the effectiveness of radiotherapy.

DNA damage responses (DDR), which can reduce cellular death, play a large and necessary role in tumor therapy (Lin et al., 2012). Ataxia telangiectasia mutated (ATM) kinase is a critical DDR element which can mutate and result in an autosomal recessive genetic disease termed Ataxia Telangiectasia (A-T). Patients who have A-T are extremely sensitive to radiotherapy, and thus are prefect subjects t receiving molecular radiosensitization. Quercetin (QT) is a major flavonoid, a class of secondary metabolic products of plants, which is used as a Chinese traditional medicine (Benkovic et al., 2009) in the treatment of asthma, as an anti-allergen, for lowering blood pressure and in treatment of tumors. Previously, it has been reported that treatment with QT can significantly increase tumor radiosensitivity by inhibiting ATM mediated pathways both *in vitro* and *in vivo*. When used systemically, natural QT is considered a radioprotective agent (Jiang et al., 2018). However, QT has poor tumor targeting ability and low water solubility which limits its systemic use during the treatment of cancers (Ma et al., 2019).

Very recently, the utilization of cellular membranes for surface functionalization of nanomaterials presents a novel method, which offers a unique advantage of a complete copy of the antigenic structure and function from cells to nanoparticles (Fang et al., 2018). Based on this, a series of cell membrane-camouflaged nanoparticles were developed and showed desirable features inherited from source cells. Nanoparticles coated with tumor cell membranes are considered to have good tumor tissue targeting and long circulating ability *in vivo* (Xie W. et al., 2019). However, there has been no research on the application of biomimetic nanoparticles for QT delivery.

In the present study, we designed a novel theranostic system CQM which consisted of a mesoporous silica nanoparticle (MSN) supported QT coated cancer cell membrane (CM) which has prolonged circulation in blood, improved tumor targeting ability, effective drug release capabilities, and strong anti-cancer effects. Mesoporous silica nanoparticles have good drug delivery characteristics due to their large surface area (>1,000 m^2^ g^−1^), high pore volume and tunable pore sizes (2–20 nm) (Ma et al., 2020). In addition, the surface of MSNs is easily loaded with hydrophobic drugs such as QT. Under acidic conditions, QT can be rapidly released from CQM and can therefore be specifically accumulated, and in combination with radiotherapy, can promote cell apoptosis. We investigated the novel theranostic system CQM *in vivo* and *in vitro* and observed that the system possessed outstanding tumor targeting ability and radio sensitivity which promotes tumor apoptosis.

## Results and Discussion

### Characterization of CMC NPs

**Figure 1:**
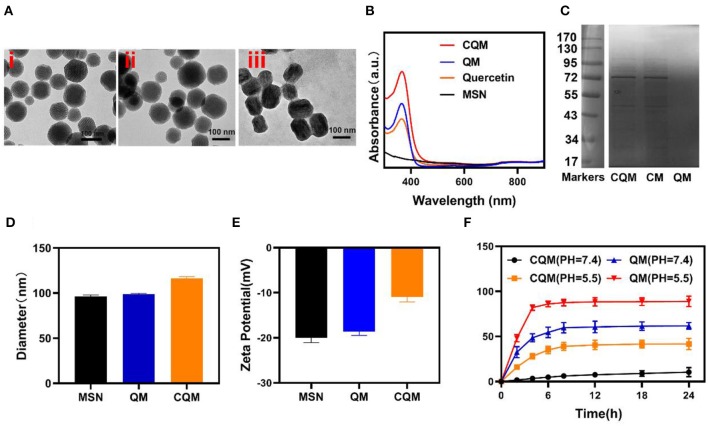
Characterization of CQM nanoparticles. **(A)** TEM image of (i) MSNs, (ii) QM, and (iii) CQM. **(B)** Absorbance spectra of CQM, QM, QT, and MSNs. **(C)** SDS-PAGE protein analysis of CQM, CM, and QM. **(D)** Hydrodynamic diameter and **(E)** Zeta potential of MSN, QM, and CQM. **(F)** QT release profiles under different conditions.

MSN NPs were successfully synthesized and loaded with QT through mechanical mixing. Transmission electron microscopy (TEM) images demonstrated that QT-loaded MSNs measured ~100 nm in diameter. Cancer cell vesicle (CV)-coated CQM particles had a 6 nm gray outer membrane (Figure 1A). Loading of QT and the CV coating were also confirmed using dynamic light scattering (DLS) analysis, SDS-PAGE, and UV-Vis spectrometry. Furthermore, while MSNs (98.5 ± 3.4 nm) and QMs (99.2 ± 4.8 nm) were similar in size, CQM (115.2 ± 6.4 nm) were larger than QM, thus indicating successful encapsulation of NPs in membrane vesicles (Figure 1D). The Zeta potential of the different particles showed similar trends (Figure 1E). In addition, CQM retained significant amounts of CM proteins (Figure 1C) and displayed characteristic peaks of QCT near 367 nm (Figure 1B). Next, we measured the release of QT from CQM NPs under different conditions. As presented in Figure 1F, under acidic conditions, QT was rapidly released from CQM and QM, whereas only small amounts were released under neutral conditions. When compared with QM, the rate of release of QT in CQM was less in acidic or neutral conditions, thus indicating less QT drug leakage.

### *In vitro* Tumor Cells Internalization

**Figure 2:**
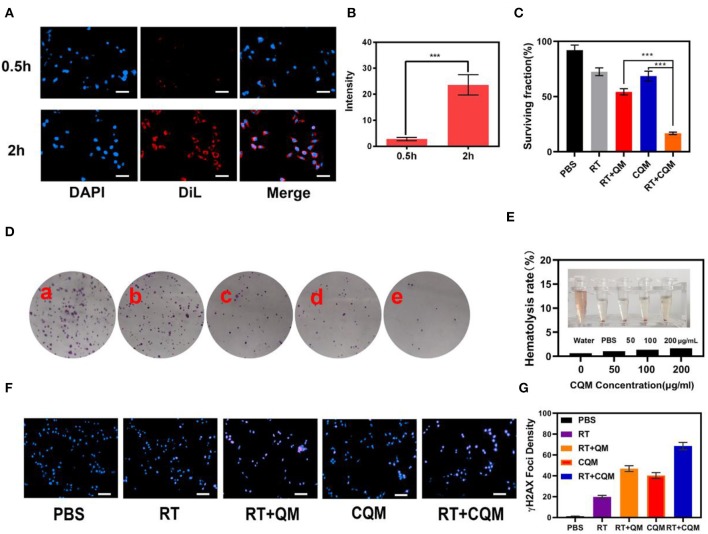
Results of *in vitro* experiments. **(A)** Fluorescence images of 4T1 cells after treatment with CQM across time. Scale bars: 50 μm. **(B)** DiL fluorescence intensity of **(A)** measured using imageJ software. **(C)** Clonogenic 4T1 cell rate of survival when co-incubated with different formulations for 12 h prior to receiving RT or not. Colonies with >50 cells were counted. **(D)** 4T1 cells treated with (a) PBS, (b) RT, (c) CQM, (d) RT+QM, or (e) RT+CQM. The MSN concentration was 200 μg/mL and the irradiation dose was 4 Gy. **(E)** Hemolysis ratio of QM at different CQM concentrations. The inset presents the corresponding hemolysis images. **(F)** Representative fluorescence images of DNA fragmentation and nuclear condensation after different treatments. DAPI and γ-H2AX were used for nuclear visualization and DNA fragmentation, respectively. Scale bars: 50 μm. **(G)** Quantitative analysis of γ-H2AX foci density (γ-H2AX foci/100 μm^−2^) for n >100 cells in each treatment group. Significant differences among groups as calculated using the Student's *t*-test. ***P < 0.005.

Following characterization of CQM nanoparticles, *in vitro* experiments were conducted. As shown in Figures 2A,B, fluorescence images of 4T1 cells following treatment with CQM was observed at different time points. CQM fluoresced Dil red and fluorescence intensity increased with incubation time, indicating that CQM is readily accumulated by tumor cells.

### *In vitro* Serum Stability and Enhanced RT

The effect of different NPs on cancer cell colony formation under X-ray irradiation was assessed. As presented in Figures 2C,D, non-irradiated PBS treated cells formed numerous and large colonies, which decreased following 4Gy irradiation. It was observed that the cancer cell survival rate differed between the CQM QM groups, thus demonstrating the cancer cell targeting capability of cancer cell membrane coated NPs. Results demonstrate that a combination of CQM and RT resulted in low colony formation and promoted cancer cell apoptosis. Serum stability of CQM in 1,640 medium containing 10% FBS was investigated. Figure 2E demonstrates that CQM was stable in blood as it caused low rates of hemolysis at all tested concentrations, thus indicating the stability of CQM in blood. Furthermore, we assessed cell DNA damage of the prepared nanoparticles (Figures 2F,G). As demonstrated in Figure 2G, no DNA damage was apparent in the control group and only some cell death was observed in the RT group. Furthermore, the CQM and RT group resulted in significant DNA damage.

### *In vivo* Pharmacokinetics and Biodistribution

**Figure 3:**
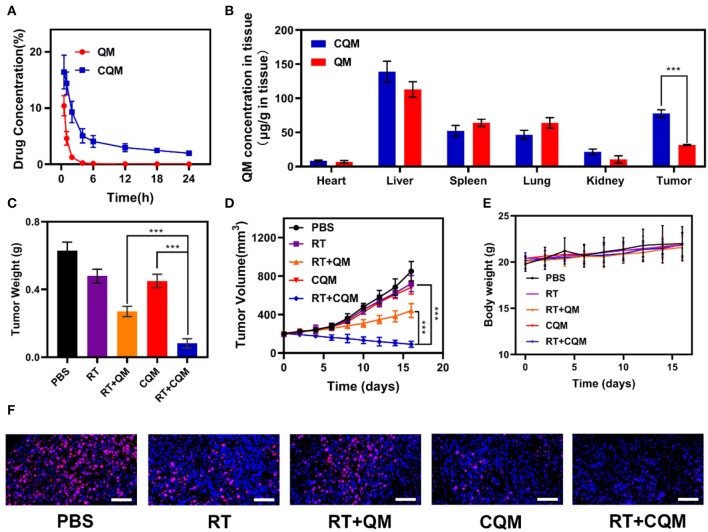
Results of *in vivo* exposures. **(A)** Changes in tumor weight following treatment. **(B)** Change in tumor-volume curves of 4T1 tumor bearing mice after treatments. **(C)** Body weight of 4T1 tumor-bearing mice as recorded every 2 days following treatment. **(D)** Pharmacokinetic behavior of QM and CQM in mice following i.v. administration at doses of 5 mg/kg of MSNs. Data are presented as mean ± SD (*n* = 3). **(E)** Quantitative analysis of QM biodistribution in tissues and tumors of tumor-bearing mice injected with QM or CQM at MSNs dose of 5 mg/kg, respectively. **(F)** Representative Ki-67 stained tumor slice images of mice following treatment. Scale bars: 100 nm. Significant differences among groups as calculated using the Student's *t*-test. ***P < 0.005.

*In vitro* experiments demonstrated that treatment with CQM when combined with radiotherapy resulted in tumor cell apoptosis. Therefore, *in vivo* pharmacokinetic experiments were conducted to investigate the effect of coated cancer membranes on retention in blood. Therefore, SD mice were administered QM and CQM or MSN *via* intravenous (i.v.) injection at doses of 5 mg/kg, respectively (Figure 3A). In comparison with the QM group, CQM exhibited enhanced blood retention, suggesting that cancer cell membrane coating prolongs evasion of the immune system MSNs. Next, we investigated biodistribution of QT in QM and CQM groups (Figure 3B). In both groups, QT distribution to the heart and kidney was low, while concentrations increased in the tumor and liver 6 h after administration. When compared with the QM group, QT distribution in the CQM group was greater, which further demonstrates the targeting ability of cancer cell membranes.

### Antitumor Efficacy *in vivo* and Histological Analysis

**Figure 4:**
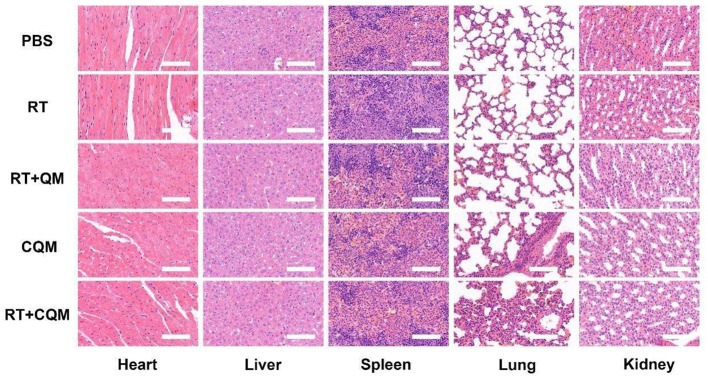
Results of histopathological analysis (H&E stained images) of the major organs; heart, liver, spleen, lung, and kidney, of mice who received various treatments 16 days post-injection under laser irradiation conditions (scale bars: 100 μm).

*In vivo* translation potential of developed NPs was investigated. Mice bearing 4T1 tumors were divided into five groups with tumor volumes of approximately 200 mm3 (n = 5 per group): (1) PBS, (2) RT, (3) RT+QM, (4) CQM, or (5) RT+CQM group. Tumor volumes were measured every 2 days using a digital caliper, and tumor weights were calculated. As presented in Figures 3C,D, tumor weight of mice treated with PBS increased rapidly throughout the experiments, whereas tumor growth in the QM of the RT group was significantly suppressed. Tumor volume and weight in the CQM of the RT group was significantly suppressed, thus indicating that combination therapy worked best. As demonstrated in Figure 3E, the body-weight of all groups developed normally indicating an absence of adverse effects. Following treatment ki-67 staining was conducted (Figure 3F), and results demonstrated that CQM + RT effectively inhibited tumor cell proliferation. Furthermore, 16 days after injection, mice were sacrificed and the heart, liver, spleen, lung, and kidney were collected and observed for histological abnormalities. Histopathological analysis of major organs (Figure 4) demonstrated that treatment with CQM did not result in noticeable pathological changes when compared with the other treatment groups, highlighting its biocompatibility.

## Conclusions

Overall, a novel drug delivery system (CQM) was designed which was composed of inner QT loaded mesoporous silica nanoparticles (MSNs) and outer cancer cell membranes (CM), which when combined with RT, promoted tumor cell apoptosis. The platform had great tumor targeting ability due to the cancer cell membrane coating. Furthermore, experimental results demonstrated that our system initiated optimal tumor cell apoptosis *in vitro* and inhibited tumor growth *in vivo*. This platform provides the basis for the development of novel radiotherapy sensitization clinical therapies.

## Data Availability Statement

The datasets generated for this study are available on request to the corresponding author.

## Ethics Statement

The animal study was reviewed and approved by Institutional Animal Care and Use Committee of Zhengzhou University (Approval number: ZDYWYJY2019018).

## Author Contributions

All authors listed have made a substantial, direct and intellectual contribution to the work, and approved it for publication.

### Conflict of Interest

The authors declare that the research was conducted in the absence of any commercial or financial relationships that could be construed as a potential conflict of interest.
